# Health-promoting behaviors mediate the relationship between eHealth literacy and health-related quality of life among Chinese older adults: a cross-sectional study

**DOI:** 10.1007/s11136-021-02797-2

**Published:** 2021-03-04

**Authors:** Shaojie Li, Guanghui Cui, Yongtian Yin, Shiyuan Wang, Xinyao Liu, Lei Chen

**Affiliations:** 1 Department of Social Medicine and Health Service Management, Xiangya School of Public Health, Central South University, Changsha, 410078 China; 2 Shandong University of Traditional Chinese Medicine, Jinan, 250355 China

**Keywords:** eHealth literacy, Health-promoting behaviors, Health-related quality of life, Mediation, Older adults

## Abstract

**Purpose:**

The aim of this study was to examine the association between eHealth literacy and health-related quality of life (HRQoL) and explore whether health-promoting behaviors mediate the association between eHealth literacy and HRQoL among Chinese older adults.

**Methods:**

An anonymous cross-sectional survey was conducted among 2300 adults aged 60 or older from Jinan, China. The eHealth Literacy Scale, Short-Form Health-Promoting Lifestyle Profile, and Short-Form Health Survey (SF-12) were used to measure eHealth literacy, health-promoting behaviors, and HRQoL. Multivariate linear regression analyses were conducted to test the association between eHealth literacy, health-promoting behaviors, and HRQoL. The mediation analyses, composed of PROCESS analysis and bootstrapping method, were preformed to test both total (*c*), direct (*c'*), and indirect effects (a*b) of eHealth literacy on HRQOL through health-promoting behaviors.

**Results:**

Regression analyses indicated that eHealth literacy (*B* = 0.487, *p* < 0.001) was significantly positively associated with health-promoting behaviors, and health-promoting behaviors (*B* = 0.257, *p* < 0.001) were associated with HRQoL. The mediation analyses indicated that eHealth literacy had a significant direct (*c'* = 0.183, *p* < 0.001) and indirect effect on older adults’ HRQoL through health-promoting behaviors (a*b = 0.125, bootstrapped 95% confidence interval (*CI*) = 0.094–0.157). The indirect effect accounted for 40.6% of the total effect (*c* = 0.308, bootstrapped 95% CI 0.241–0.376) of eHealth literacy on HRQoL.

**Conclusions:**

Health-promoting behaviors mediated the association between eHealth literacy and HRQoL in Chinese older adults. The establishment of interventions focused on health-promoting behavior may be an effective way to help older adults with low eHealth literacy improve their HRQoL.

## Introduction

Health-related quality of life (HRQoL) refers to individuals’ subjective assessment of their own well-being and ability to perform physical, psychological, and social functions [1]. As a comprehensive health indicator, HRQoL not only reflects one’s physical and mental health status and life satisfaction [2] but is also regarded as an important care outcome for older adults and is widely used in medical interventions and health population surveys [3, 4]. As the body ages, older adults’ health and functioning declines, and they are prone to developing chronic diseases [5]; therefore, it is vital to maintain and promote older adults’ HRQoL. HRQoL in older adults is associated with several factors, including gender, education level, income, and other general demographic data [6]. However, it is difficult to design and implement relevant interventions because these sociodemographic characteristics are difficult to change. As a result, people have begun to look for those factors that can be changed, and health literacy has come into focus [7].

Health literacy refers to an individual’s ability to acquire, process, and understand basic health information and services and use them to make appropriate health decisions [8]. Currently, there is some evidence that improvements in health literacy contribute to improved HRQoL [9, 10]. A systematic review showed that low health literacy is associated with poor health outcomes [11]; however, it is not clear whether this relationship can be generalized to eHealth literacy. eHealth literacy is an extension of the concept of health literacy [12] and refers to the ability to seek, find, understand, and appraise health information from electronic sources and apply the knowledge gained to address or solve a health problem [13]. It should be noted that although the concept of eHealth literacy is derived from health literacy, this does not mean that eHealth literacy is part of health literacy. Previous studies found that the correlation between health literacy and eHealth literacy is moderate [14], and eHealth literacy is affected by a higher level of health literacy [15]. Therefore, health literacy and eHealth literacy need to be distinguished in research. Unlike health literacy, eHealth literacy emphasizes the ability to obtain and use relevant health information through electronic media. In other words, individuals with low health literacy can still benefit their own health if they have the ability to use electronic media to search for and understand online health information. Given the rapid development of electronic communication technology, improving eHealth literacy may be able to help improve the health status of older adults with low health literacy.

With the development of Internet information technology, a large amount of health information can be transmitted through the Internet. China’s statistical report on Internet development showed that the proportion of Internet users among Chinese older adults is increasing, with the number of Internet users aged 50 and above rising from 7.3% in June 2014 [16] to 13.6% in June 2019 [17]. This indicates that older adults are increasingly beginning to use the Internet to search for information. However, although the Internet can provide resources for individuals seeking knowledge about health conditions and treatment, this does not mean that people can always properly use these resources to solve health problems [13]. One study found that even groups with sophisticated Internet technology still lacked the ability to assess the quality of online health information [18]. Therefore, in the face of the growing number of elderly Internet users in China, investigating older adults’ eHealth literacy could not only help promote their use of Internet health information but could also have important public health implications regarding their improved health.

Research on eHealth literacy and health outcomes is clearly in its early stages [19]. Only a few studies have examined the relationship between eHealth literacy and HRQoL in patients with chronic diseases [20], and as far as we know, no study has focused on the potential mechanism for the association between eHealth literacy and HRQoL. The Electronic Health-Integrated Use Model indicates that individuals with low eHealth literacy lack the motivation to use online health information to improve their health and that they believe they cannot use online health information to improve health behaviors and promote health [21]. This suggests that health behaviors may be a potential mediating factor in the association between eHealth literacy and HRQoL. Previous studies have found also that eHealth literacy had a positive effect on personal health-promoting behaviors [22]. Health-promoting behavior includes self-actualization, health responsibility, exercise, nutrition, interpersonal support, and stress management [23] and has been recognized as an important element for preventing and controlling diseases, which maintains and promotes HRQoL [24]. Studies have found that health-promoting behaviors can mediate the relationship between individual self-perception (such as perceived stress and self-efficacy) and health outcomes (such as mental health and HRQoL) [25, 26]. Considering that eHealth literacy is closely related to health-promoting behaviors, and health-promoting behaviors can promote HRQoL, we have reason to assume that the relationship between eHealth literacy and HRQoL may be mediated by health-promoting behaviors.

Therefore, the purpose of this study was to examine the association between eHealth literacy and HRQoL and explore whether health-promoting behaviors mediate the association between eHealth literacy and HRQoL among older adults. The results could be utilized as a reference for designing future eHealth literacy intervention programs and methods to improve overall HRQoL for older adults.

## Methods

### Study participants and sampling procedure

**Fig. 1:**
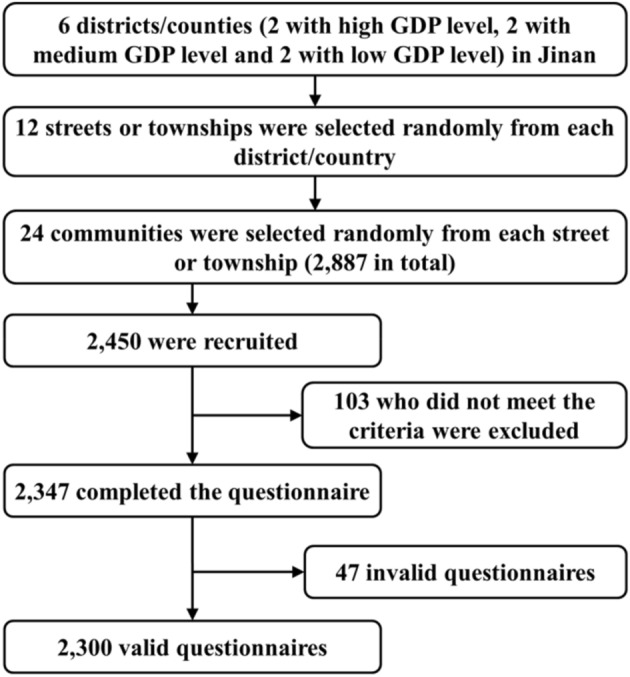
Participant enrollment procedure

From June to August 2020, a cross-sectional population-based study was conducted with a multistage sampling in Jinan City, Shandong Province, China. At the end of 2019, Jinan’s 10 districts and two counties had a total population of 8.909 million; older adults comprised 21% of the population. Figure 1 shows the sampling procedure. In the first stage, Jinan’s 12 districts/counties in 2019 were categorized into three levels according to their gross domestic product (GDP) per capita: high GDP (four districts/counties), medium GDP (four districts/counties), and low GDP (four districts/counties), and two districts/counties were randomly selected from each category for a total of six districts/counties. In the second stage, two streets or townships were randomly selected from the six districts/counties, respectively, and a total of 12 streets or townships were selected. In the third stage, two communities from the 12 streets or townships were selected, respectively. Finally, a total of 24 communities were selected.

After identifying the communities, we contacted the managers of the communities by phone and introduced them to the research content, purpose, and value. With their help, we obtained a list of names and phone numbers of older adults (2887 people in total). We used mobile phone text messages to send recruitment information, including research purpose, content, survey time, and survey site (community public service center) to all older adults. Considering that the elderly are a vulnerable group, we required their family members (children or spouses) to accompany them when participating in the survey. A total of 2450 people responded to the information and went to the survey site to participate. Before starting the survey, uniformly trained investigators screened the research participants according to the inclusion and exclusion criteria. The inclusion criteria were as follows: age ≥ 60 years; good language communication and cognitive judgment ability (self-reported); the ability to complete basic measures (self-reported); the ability to surf the Internet (self-reported); and the ability to provide voluntary informed consent and follow the study procedure. The exclusion criteria were as follows: patients with severe and terminal diseases (as reported by family members); severe cognitive impairment such as dementia and confusion (as reported by family members); and hearing and visual impairments (as reported by family members). Regarding the inclusion and exclusion criteria, age ≥ 60 years was set according to the classification criteria for the elderly in developing countries proposed by the World Health Organization [27]. The ability to surf the Internet was determined based on one of our research variables, eHealth literacy, because eHealth literacy necessitates the ability to use the Internet. The other criteria were determined to ensure that participants would not be unable to complete the survey due to physical health problems.

In the end, 103 people who did not meet the criteria were excluded, and 2347 people completed the questionnaire and signed a written informed consent form. All questionnaires were self-reported. In addition, for some participants who were unable to complete the questionnaire due to illiteracy, the investigator assisted them in completing the questionnaire, that is, the investigator verbally asked the participant each question, the participant answered, and the investigator wrote the answer on the questionnaire. As participants refused to answer some questions in the survey, 47 questionnaires were missing over 10% of content. After excluding these missing content questionnaires, the final sample comprised 2300 valid questionnaires. The study was approved by the Medical Ethics Committee of Central South University (CTXY-150002-7).

### Measurements

#### eHealth literacy

eHealth literacy was measured using the eHealth Literacy Scale (eHEALs), which was developed by Norman and Skinner [28]. This scale is an eight-item instrument designed to assess subjective eHealth literacy. The eight items are rated on a five-point Likert scale (1–5 points), and total scores range from 8 to 40 points. A higher score indicates a better eHealth literacy. The instrument has been validated for use in Chinese populations, and its reliability has been reported with a Cronbach’s alpha 0.83 for the total scale [29]. In this study, Cronbach’s alpha was 0.98.

#### Health-promoting behaviors

Health-promoting behaviors were measured using the short-form Health-Promoting Lifestyle Profile (HPLP). It was simplified and revised on the basis of the 48-item Health-Promoting Lifestyles Profile [23] by Wei et al. [30]. The scale has 24 items, including six dimensions of self-actualization, health responsibility, exercise, nutrition, interpersonal support, and stress management. Each dimension is evaluated with 4 items. Each item uses a four-point Likert scoring method (1–4 points). The total score is obtained by adding the scores of the six dimensions, with a range from 24 to 96, and higher scores indicate better health-promoting behaviors. The Cronbach’s alpha of the scale in this study was 0.94.

#### Health-related quality of life

HRQoL was measured using the Short-Form Health Survey (SF-12). This survey has been widely applied to groups of older adults in China [6]. The scale comprises 12 items that measure physical functioning, role physical, role emotional, bodily pain, general health, vitality, social functioning, and mental health. In this study, the total score of SF-12 was used to assess HRQoL. According to the score criteria [31], the total score was transformed into standard scores, ranging from 0 to 100, with higher scores indicating better HRQoL. Cronbach’s alpha in this study was 0.72.

#### Covariates

Based on previous research on Chinese older adults [32–35], we used sociodemographic characteristics and activities of daily living (ADL), frequently used in previous studies, as covariates. All covariates were collected using a self-reported paper questionnaire. The sociodemographic characteristics included age, gender, residence, education level, marital status, and average monthly family income. Among these factors, age was divided into three categories, 60–69, 70–79, and ≥ 80, and education level was divided into four categories, primary school and below, junior high school, high school, and university and above. Marital status was described as “married” or “unmarried.” Average monthly family income was assessed by dividing the annual family income (self-reported) by 12 months. Then, the respondents were ranked from lowest to highest by their average monthly family income and divided into three groups according to their 33% and 66% percentiles. The lowest income had an income below 5000 RMB, and the second group was from 5000 to 11,000 RMB, and the highest group had 11,000 RMB above. ADL were assessed by the Barthel Index [36]. The scale comprised 10 items, including feeding, bathing, grooming, dressing, bowels control, bladder control, toilet use, transfers (bed to chair and back), mobility (on level surfaces), and stair climbing. Scoring was based on how much help the older adult needed. The total score was from 0 to 100 points; the higher the score, the higher the ability of the elderly to perform ADL. In this study, Cronbach’s alpha was 0.94.

### Statistical analysis

Data are presented as *n* (%) for categorical variables and mean ± standard deviation for numerical variables. Comparisons of mean scores of eHEALs, HPLP, and HRQoL between different participant characteristics were made by student’s t test and one-way analysis of variance (ANOVA). Two separate multivariate linear regression models were used to test the association between eHealth literacy, health-promoting behaviors, and HRQoL (all of which were treated as continuous variables).

**Fig. 2:**
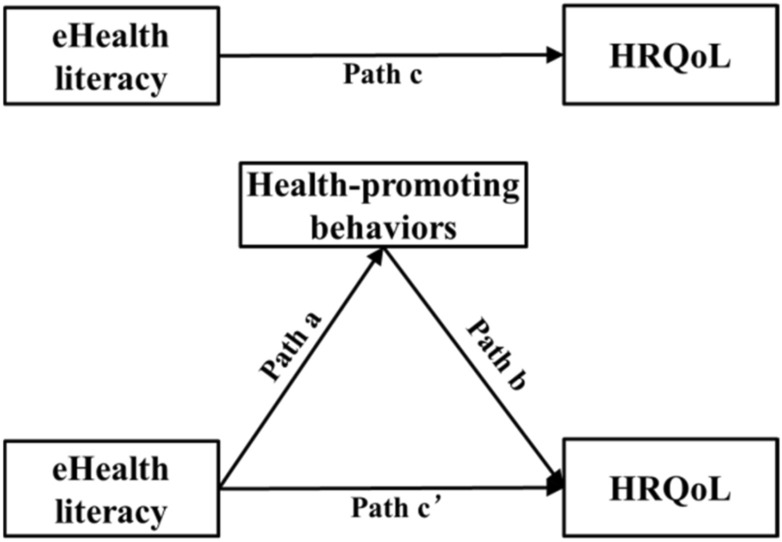
The conceptual mediation model eHealth literacy, health-promoting behaviors, and HRQOL

Figure 2 shows the theoretical model used to assess the mediating effect of health-promoting behaviors in the relationship between eHealth literacy and HRQoL; among them, *c'* refers to the direct effect of eHealth literacy on HRQoL, and the product of *a* and *b* (a*b) refers to the indirect effect of eHealth literacy on HRQoL through health-promoting behaviors. And *c* represents the total effect of eHealth literacy on HRQoL, namely *c'* + a*b. The mediation analysis was applied using the SPSS PROCESS macro developed by Hayes [37]. PROCESS macro is based on ordinary least-squares (OLS) regression and incorporates a nonparametric bootstrapping procedure for assessing mediation [37], which is widely used in current mediating effects testing [38–40]. In the PROCESS macro, we entered the eHEALS total score as the prediction variable, the HPLP total score as the mediator variable, and the HRQoL score as the outcome variable, and bootstrapping procedures were set to 10,000 samples and were used to test the estimated mediating effect (a*b). In all analyses, sociodemographic characteristics (age, gender, residence, education level, marital status, and average monthly family income) and ADL were used as covariates to overcome potential confounding effects. All statistical analyses were performed using SPSS 25.0 (IBM SPSS Statistics, Armonk, NY, USA).

## Results

### Participant characteristics

A total of 2300 Chinese older adults completed the survey, and mean participant age was 70.3 ± 6.4 years, with a range from 60 to 97 years old. The participants’ sociodemographic characteristics were as follows: 1201 (52.2%) were male, 1683 (73.2%) lived in rural area, 1410 (61.3%) completed primary school or below, and 1749 (76.0%) were married. The average monthly family income ranged from 752 to 35,754 RMB (mean = 7421.96, SD = 3685.14). The ADL mean score was 95.44 ± 14.32, with a range from 0 to 100. The mean scores of eHEALS, HPLP, and HRQoL were 18.60 ± 9.76, 63.00 ± 13.43, and 59.23 ± 16.31, respectively. Further participant characteristics and the status of eHealth literacy, health-promoting behaviors, and HRQoL by different participant characteristics are presented in Table 1.

**Table 1:** The status of eHealth literacy, health-promoting behaviors, and HRQoL by different characteristics

Variables	*N* (%)	eHEALs	*t/F*	*p*-value	HPLP	*t/F*	*p*-value	HRQoL	*t/F*	*p*-value
Total		18.60 ± 9.76			63.00 ± 13.43			59.23 ± 16.31		
Scores range		8.00–40.00			24.00–96.00			10.94–100.00		
Age(years)			5.032	0.007		6.399	0.022		19.816	< 0.001
60–69	1135 (49.3)	19.19 ± 9.36			63.19 ± 12.50			60.64 ± 15.74		
70–79	953 (41.4)	18.20 ± 10.08			63.46 ± 14.11			58.92 ± 16.90		
≥ 80	212 (9.2)	17.22 ± 10.14			59.89 ± 14.70			53.08 ± 15.10		
Gender			3.985	< 0.001		0.820	0.413		4.204	< 0.001
Male	1201 (52.2)	19.37 ± 9.640			63.22 ± 13.681			60.59 ± 16.54		
Female	1099 (47.8)	17.76 ± 9.815			62.76 ± 13.153			57.74 ± 15.92		
Residence			12.641	< 0.001		6.158	< 0.001		2.039	0.049
Urban area	617 (26.8)	22.71 ± 9.91			65.82 ± 14.63			60.38 ± 17.16		
Rural area	1683 (73.2)	17.10 ± 9.25			61.96 ± 12.81			58.81 ± 15.97		
Education level			77.207	< 0.001		44.755	< 0.001		9.294	< 0.001
Primary school or below	1410 (61.3)	16.63 ± 8.96			60.72 ± 12.30			57.98 ± 16.26		
Junior high school	590 (25.7)	20.11 ± 9.51			65.32 ± 13.41			60.24 ± 15.56		
High school	233 (10.1)	23.72 ± 10.75			68.04 ± 15.25			63.17 ± 17.91		
University and above	67 (2.9)	28.93 ± 9.07			72.84 ± 16.90			62.92 ± 14.90		
Marital status			4.486	< 0.001		6.391	< 0.001		5.647	< 0.001
Married	1749 (76.0)	19.11 ± 9.75			63.99 ± 13.39			60.30 ± 16.21		
Unmarried	551 (24.0)	16.98 ± 9.59			59.83 ± 13.06			55.83 ± 16.16		
Average monthly family income (RMB)			42.941	< 0.001		40.583	< 0.001		2.248	0.106
< 5000	783 (34.0)	16.25 ± 9.07			59.99 ± 12.83			58.31 ± 16.83		
5000–11,000	753 (32.7)	18.88 ± 9.22			63.02 ± 12.54			59.36 ± 15.93		
> 11,000	764 (33.2)	20.74 ± 10.41			66.04 ± 14.19			60.05 ± 16.10		

Data are presented as mean ± SD

*eHEALs* eHealth Literacy Scale, *HPLP* Health-Promoting Lifestyle Profile, *HRQoL* Health-Related Quality of Life

### Association among eHealth literacy, health-promoting behaviors, and HRQoL

Table 2 presents the results of the multivariate linear regression model of association among eHealth literacy, health-promoting behaviors, and HRQoL. After controlling for covariates such as age, gender, residence, education level, marital status, average monthly family income, and ADL, Model 1 showed that eHealth literacy (*B* = 0.487, *p* < 0.001) was significantly positively associated with health-promoting behaviors, and Model 2 showed that eHealth literacy (*B* = 0.183, *p* < 0.001) and health-promoting behaviors (*B* = 0.257, *p* < 0.001) were both associated with HRQoL.

**Table 2:** Multivariate liner regression analysis of association among eHealth literacy, health-promoting behaviors, and HRQoL

Predictors	Model 1 (HPLP)				Model 2 (HRQoL)			
	*B*	95% CI	SE	*p-*value	*B*	95% CI	SE	*p-*value
Age	0.426	(− 0.354, 1.205)	0.398	0.285	− 1.259	(− 2.215, − 0.304)	0.487	0.010
Gender	0.988	(− 0.013, 1.989)	0.510	0.053	− 2.182	(− 3.409, − 0.956)	0.625	< 0.001
Residence	0.837	(− 0.409, 2.084)	0.636	0.188	− 0.088	(− 1.615, 1.439)	0.779	0.910
Education level	1.900	(1.189, 2.612)	0.363	< 0.001	0.200	(− 0.676, 1.077)	0.447	0.654
Marital status	− 2.599	(− 3.789, − 1.408)	0.607	< 0.001	− 1.214	(− 2.678, 0.250)	0.747	0.104
Average monthly family income	1.412	(0.778, 2.046)	0.323	< 0.001	− 0.741	(− 1.520, 0.038)	0.397	0.062
ADL	0.094	(0.059, 0.129)	0.018	< 0.001	0.317	(0.274, 0.360)	0.022	< 0.001
eHealth literacy	0.487	(0.433, 0.541)	0.028	< 0.001	0.183	(0.113, 0.254)	0.036	< 0.001
HPLP					0.257	(0.207, 0.307)	0.026	< 0.001

*B* unstandardized coefficient

*CI* confidence interval, *SE* standardized error, *HPLP* Health-Promoting Lifestyle Profile, *HRQoL* Health-Related Quality of Life; Model 1 used the HPLP total score as the dependent variable; Model 2 used the HRQoL total score as the dependent variable

### Mediation model analysis

**Fig. 3:**
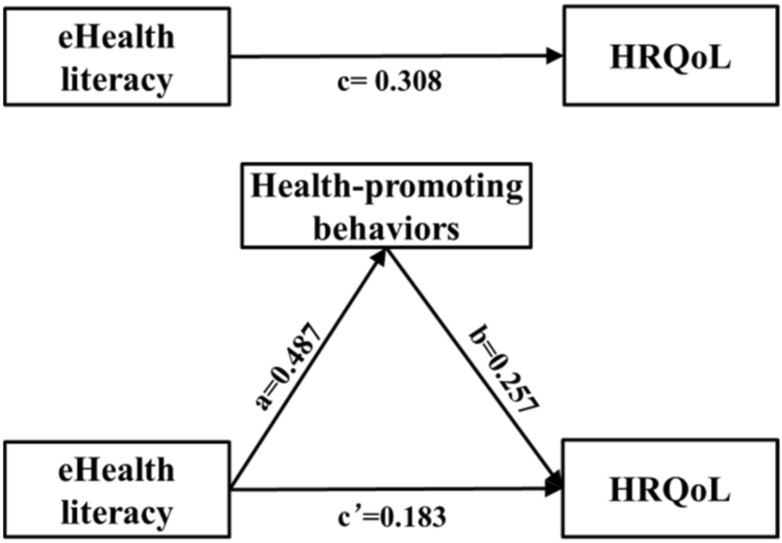
Model of the mediating effect of health-promoting behaviors on the association between eHealth literacy and HRQoL

Table 3 and Fig. 3 present the mediation analysis result based on the PROCESS macro. The results indicate that the indirect effect (a*b) value of health-promoting behaviors in the association between eHealth literacy and HRQoL was 0.125, with a 95% confidence interval (CI) from 0.094 to 0.157. The bootstrapped 95% CI did not include 0 to indicate that the mediating effect is statistically significant. The mediating effect accounted for 40.6% of the total effect (*c* = 0.308, bootstrapped 95% CI 0.241–0.376). These results reveal that health-promoting behaviors mediated the association between eHealth literacy and HRQoL.

**Table 3:** Testing the mediation effect of health-promoting behaviors analysis based on PROCESS

	*B*	95% CI	SE	*p*-value
Total effect, *c*	0.308	(0.241, 0.376)	0.034	< 0.001
Direct effect, *c*ʹ	0.183	(0.113, 0.254)	0.036	< 0.001
Indirect effect, a*b	0.125	(0.094, 0.157)	0.016	< 0.001
Ratio of indirect to total effect mediated (a*b/*c*)	40.6%			

*B* unstandardized coefficient, *CI* confidence interval, *SE* standardized error

## Discussion

The current study explored the association between eHealth literacy and HRQoL in older adults and examined the mediating role of health-promoting behaviors. Our results showed that eHealth literacy was positively associated with HRQoL, and health-promoting behaviors mediated the association between eHealth literacy and HRQoL in Chinese older adults. They provide evidence to support the idea that eHealth literacy may influence HRQoL through health-promoting behaviors. These findings may contribute to the design of improvement strategies for older adults’ HRQoL in the context of the rapid development of Internet health information platforms.

The mean score of Chinese older adults’ eHealth literacy was 18.6 points, which was lower than previous studies of American older adults (mean score was 29.1) [41]. This suggests that the Chinese older adults’ eHealth literacy is low, and Chinese government should pay more attention to improving older adults’ eHealth literacy. Previous studies have shown that younger age was associated with higher eHealth literacy [42], which is consistent with our research. This may be because as age increases, cognitive ability gradually declines, resulting in decreased ability to learn and understand online health knowledge. We also found that men’s eHealth literacy was higher than women’s, which is contrary to previous research on health literacy [43]. This may be related to men’s social media habits, as a previous study suggested that men were actually more likely than women to use social media to research health and wellness issues [44]. Additionally, we found that older adults with lower eHealth literacy level were more likely to report factors related to a lower socioeconomic status, such as living in rural areas, receiving less education, being unmarried, and having a lower income. This may be related to low availability of health-related information and resources for people with lower socioeconomic status.

The results of the study showed that the differences in health-promoting behaviors scores between older adults of different ages, residence, education level, marital status, and average monthly family income were statistically significant. In addition, there were also significant differences in HRQOL scores among the older adults with different gender, age, residence, education level, and marital status. These results are similar to those of previous studies [45–48]. These findings suggest that more health interventions should be provided to older adults who are elderly, women, living in rural areas, unmarried, and those with lower education level and family income.

Our study found that eHealth literacy was positively associated with HRQoL. This indicates that older adults with poor eHealth literacy were more likely to have limited physical health and poor mental health. Previous studies have widely discussed the impact of health literacy on HRQoL [49]. Studies have found that eHealth literacy was related to the HRQoL of patients with chronic obstructive pulmonary disease [20]. However, there were large sociodemographic and health differences between the sample of patients with chronic obstructive pulmonary disease and our sample of healthy older adults; therefore, the relationship between eHealth literacy and HRQoL cannot be directly inferred for the elderly population. As far as we know, our study is the first attempt to investigate the relationship between eHealth literacy and HRQoL of older adults and explore the mediating role of health-promoting behaviors.

The mediation effect may be explained by Andersen’s Behavioral Model of Health Services Use. The model highlights that health behaviors are correlated with individual characteristics and health outcomes, and individual characteristics can affect health outcomes through health behaviors [50]. In this study, eHealth literacy may be regarded as an individual characteristic because it reflects the individual’s perception of the collection, evaluation, and application of online health information. According to this model, it can be inferred that older adults with higher eHealth literacy may have a stronger subjective initiative to adopt health-promoting behaviors, which in turn helps improve HRQoL. Specifically, older adults with higher eHealth literacy may have more confidence in using online health information to promote health [51] and are more likely to obtain health promotion resources and knowledge, which may help increase health management awareness and their adoption of health-promoting behaviors, which would improve HRQoL.

There are several limitations that need to be noted. First, this study adopted a cross-sectional study design, which means that the causal relationship between eHealth literacy, health-promoting behaviors, and HRQoL cannot be determined. Previous study showed that due to differing degrees of individual exposure to eHealth services, eHealth literacy varies widely among individuals [52]. This could mean that HRQoL may in turn affect eHealth literacy. A study in the United States found that individuals with poor health are more likely to use the Internet to seek health information [53]. Given this, more longitudinal studies or intervention studies are needed to determine causal relationships among eHealth literacy, health-promoting behaviors, and HRQoL in the future. Second, since we only selected one city in China for the survey, the study population is not representative of the general Chinese older population, so the results may not be generalizable. Further, although we used a strict randomization method to select samples, some participants still did not agree to participate in this study, which may have caused selection bias. Third, the current mediation model was based on previous research experience and lacked sufficient theoretical support. Finally, this study used self-report measures, which may have led to some information bias.

## Conclusions

Poor eHealth literacy was associated with poor HRQoL of older adults, and health-promoting behaviors mediated the association between eHealth literacy and HRQoL. The results indicate that taking older adults’ eHealth literacy levels into account when formulating health education and promotion programs for older adults could be beneficial, especially when the expected outcome is to improve HRQoL. Establishing health-promoting behavior interventions may be an effective way to help older adults with low eHealth literacy improve their HRQoL.

## References

[1] Wang H-M, Beyer M, Gensichen J, Gerlach FM (2008). Health-related quality of life among general practice patients with differing chronic diseases in Germany: Cross sectional survey. BMC Public Health.

[2] Proctor C, Maltby J, Linley PA (2011). Strengths use as a predictor of well-being and health-related quality of life. Journal of Happiness Studies.

[3] Karimi M, Brazier JJP (2016). Health, health-related quality of life, and quality of life: What is the difference?. PharmacoEconomics.

[4] Martinez-Martin P (2017). What is quality of life and how do we measure it? Relevance to Parkinson's disease and movement disorders. Movement Disorders.

[5] Ferris R, Blaum C, Kiwak E, Austin J, Esterson J, Harkless G, Oftedahl G, Parchman M, Van Ness PH, Tinetti ME (2018). Perspectives of patients, clinicians, and health system leaders on changes needed to improve the health care and outcomes of older adults with multiple chronic conditions. Journal of aging and health.

[6] Ma XG, McGhee SM (2013). A cross-sectional study on socioeconomic status and health-related quality of life among elderly Chinese. British Medical Journal Open.

[7] Nutbeam D (2000). Health literacy as a public health goal: A challenge for contemporary health education and communication strategies into the 21st century. Health Promotion International.

[8] Berkman ND, Davis TC, McCormack L (2010). Health literacy: What is it?. Journal of Health Communication.

[9] Halverson JL, Martinez-Donate AP, Palta M, Leal T, Lubner S, Walsh MC, Schaaf Strickland J, Smith PD, Trentham-Dietz A (2015). Health literacy and health-related quality of life among a population-based sample of cancer patients. Journal of Health Communication.

[10] Gonzalez-Chica DA, Mnisi Z, Avery J, Duszynski K, Doust J, Tideman P, Murphy A, Burgess J, Beilby J, Stocks N (2016). Effect of health literacy on quality of life amongst patients with ischaemic heart disease in Australian General Practice. PLoS ONE.

[11] Chesser AK, Keene Woods N, Smothers K, Rogers N (2016). Health literacy and older adults: A systematic review. Gerontology Geriatric Medicine.

[12] Luo YF, Yang SC, Chen A-S, Chiang C-H (2018). Associations of ehealth literacy with health services utilization among college students: Cross-sectional study. Journal of Medical Internet Research.

[13] Norman CD, Skinner HA (2006). eHealth literacy: Essential skills for consumer health in a networked world. Journal of Medical Internet Research.

[14] Neter E, Brainin E, Baron-Epel O (2015). The dimensionality of health literacy and eHealth literacy. European Health Psychologist.

[15] Del Giudice P, Bravo G, Poletto M, De Odorico A, Conte A, Brunelli L, Arnoldo L, Brusaferro S (2018). Correlation between eHealth literacy and health literacy using the eHealth Literacy Scale and real-life experiences in the health sector as a proxy measure of functional health literacy: cross-sectional web-based survey. Journal of Medical Internet Research.

[16] CNNIC (2014) The 34th Survey Report on Internet Development in China. http://www.cnnic.net.cn/hlwfzyj/hlwxzbg/hlwtjbg/201902/P020190318523029756345.pdf. Accessed 21 July 2014

[17] CNNIC (2019) The 43th Survey Report on Internet Development in China. http://www.cnnic.net.cn/hlwfzyj/hlwxzbg/hlwtjbg/201902/P020190318523029756345.pdf. Accessed 30 August 2019

[18] Feufel MA, Stahl SF (2012). What do web-use skill differences imply for online health information searches?. Journal of Medical Internet Research.

[19] Neter E, Brainin E (2019). Association between health literacy, ehealth literacy, and health outcomes among patients with long-term conditions. European Psychologist.

[20] Stellefson M, Paige SR, Alber JM, Chaney BH, Chaney D, Apperson A, Mohan A (2019). Association between health literacy, electronic health literacy, disease-specific knowledge, and health-related quality of life among adults with chronic obstructive pulmonary disease: Cross-sectional study. Journal of Medical Internet Research.

[21] Bodie GD, Dutta MJ (2008). Understanding health literacy for strategic health marketing: eHealth literacy, health disparities, and the digital divide. Health Marketing Quarterly.

[22] Kim KA, Kim YJ, Choi M (2018). Association of electronic health literacy with health-promoting behaviors in patients with Type 2 diabetes: A cross-sectional study. Computers, Informatics, Nursing.

[23] Walker SN, Sechrist KR, Pender NJ (1987). The Health-Promoting Lifestyle Profile: development and psychometric characteristics. Nursing Research.

[24] Şenol V, Ünalan D, Soyuer F, Argün M (2014). The relationship between health promoting behaviors and quality of life in nursing home residents in Kayseri. Journal of Geriatrics.

[25] Majidi Yaychi N, Hasanzade R, Farmarzi M, Homayoni A (2019). Mediating role of health promoting behaviors on the relationship between self-efficacy and mental health in adolescent. Education Community Health.

[26] Seo EJ, Ahn J-A, Hayman LL, Kim C-J (2018). The association between perceived stress and quality of life in university students: The parallel mediating role of depressive symptoms and health-promoting behaviors. Asian Nursing Research.

[27] World Health O. (2015). World report on ageing and health. World Health Organization,

[28] Norman CD, Skinner HA (2006). eHEALS: The ehealth literacy scale. Journal of Medical Internet Research.

[29] Chang A, Schulz P (2018). The measurements and an elaborated understanding of Chinese eHealth literacy (C-eHEALS) in chronic patients in China. International Journal of Environmental Research Public HEALTH.

[30] Wei M-H, Lu C-M (2005). Development of the short-form Chinese Health-Promoting Lifestyle Profile. Journal of health Education.

[31] Ware JE, Keller SD, Kosinski M (1995). SF-12: How to score the SF-12 physical and mental health summary scales.

[32] Wu M, Yang Y, Zhang D, Zhao X, Sun Y, Xie H, Jia J, Su Y, Li Y (2018). Association between social support and health-related quality of life among Chinese rural elders in nursing homes: The mediating role of resilience. Quality of Life Research.

[33] Yang Y, Wang S, Chen L, Luo M, Xue L, Cui D, Mao Z (2020). Socioeconomic status, social capital, health risk behaviors, and health-related quality of life among Chinese older adults. Health and Quality of Life Outcomes.

[34] You XY, Zhang YL, Zeng JF, Wang CJ, Sun HP, Ma QH, Ma YN, Xu Y (2019). Disparity of the Chinese elderly's health-related quality of life between urban and rural areas: A mediation analysis. British Medical Journal Open.

[35] Li J, Xu X, Sun J, Cai W, Qin T, Wu M, Liu H (2020). Activities of daily living, life orientation, and health-related quality of life among older people in nursing homes: A national cross-sectional study in China. Quality of Life Research.

[36] Mahoney FI, Barthel DW (1965). Functional evaluation: The Barthel Index. Maryland State Medical Journal.

[37] Hayes AF (2017). Introduction to mediation, moderation, and conditional process analysis: A regression-based approach.

[38] De Gucht V (2015). Illness perceptions mediate the relationship between bowel symptom severity and health-related quality of life in IBS patients. Quality of Life Research.

[39] Kristofferzon M-L, Engström M, Nilsson A (2018). Coping mediates the relationship between sense of coherence and mental quality of life in patients with chronic illness: A cross-sectional study. Quality of Life Research.

[40] Kwok JYY, Choi EPH, Chau PH, Wong JYH, Fong DYT, Auyeung M (2020). Effects of spiritual resilience on psychological distress and health-related quality of life in Chinese people with Parkinson’s disease. Quality of Life Research.

[41] Stellefson M, Paige SR, Tennant B, Alber JM, Chaney BH, Chaney D, Grossman S (2017). Reliability and validity of the telephone-based eHealth literacy scale among older adults: Cross-sectional survey. Journal of Medical Internet Research.

[42] Tennant B, Stellefson M, Dodd V, Chaney B, Chaney D, Paige S, Alber J (2015). eHealth literacy and Web 2.0 health information seeking behaviors among baby boomers and older adults. JMIR.

[43] Robinson S, Moser D, Pelter MM, Nesbitt T, Paul SM, Dracup K (2011). Assessing health literacy in heart failure patients. Journal of Cardiac Failure.

[44] Elkin, N. (2008). How America searches: Health and wellness. Opinion Research Corporation: iCrossing 1–17.

[45] Song D, Yu DS, Li PW, He G, Sun Q (2019). Correlates of health-related quality of life among Chinese older adults with mild cognitive impairment. Clinical Interventions in Aging.

[46] Bilgili N, Arpacı F (2014). Quality of life of older adults in Turkey. Archives of Gerontology and Geriatrics.

[47] Harooni J, Hassanzadeh A, Mostafavi F (2014). Influencing factors on health promoting behavior among the elderly living in the community. Journal of Education and Health Promotion.

[48] Rakhshani T, Shojaiezadeh D, Lankarani KB, Rakhshani F, Kaveh MH, Zare N (2014). The association of health-promoting lifestyle with quality of life among the Iranian elderly. Iranian Red Crescent Medical Journal.

[49] Panagioti M, Skevington SM, Hann M, Howells K, Blakemore A, Reeves D, Bower P (2018). Effect of health literacy on the quality of life of older patients with long-term conditions: A large cohort study in UK general practice. Quality of Life Research.

[50] Andersen RM, Davidson PL, Baumeister SE, Kominski G (2014). Improving access to care in America. Changing the U.S. health care system: Key issues in health services policy and management.

[51] Heiman H, Keinki C, Huebner J (2018). eHealth literacy in patients with cancer and their usage of web-based information. Journal of Cancer Research Clinical Oncology.

[52] Lustria MLA, Smith SA, Hinnant CC (2011). Exploring digital divides: an examination of eHealth technology use in health information seeking, communication and personal health information management in the USA. Health Informatics Journal.

[53] Lee YJ, Boden-Albala B, Larson E, Wilcox A, Bakken S (2014). Online health information seeking behaviors of hispanics in New York City: A community-based cross-sectional study. Journal of Medical Internet Research.

